# Significant and Conflicting Correlation of IL-9 With *Prevotella* and *Bacteroides* in Human Colorectal Cancer

**DOI:** 10.3389/fimmu.2020.573158

**Published:** 2021-01-08

**Authors:** Elena Niccolai, Edda Russo, Simone Baldi, Federica Ricci, Giulia Nannini, Matteo Pedone, Francesco Claudio Stingo, Antonio Taddei, Maria Novella Ringressi, Paolo Bechi, Alessio Mengoni, Renato Fani, Giovanni Bacci, Camilla Fagorzi, Carolina Chiellini, Domenico Prisco, Matteo Ramazzotti, Amedeo Amedei

**Affiliations:** ^1^ Department of Clinical and Experimental Medicine, University of Florence, Florence, Italy; ^2^ Department of Biomedical, Experimental and Clinical Sciences “Mario Serio” University of Florence, Florence, Italy; ^3^ Department of Statistics, Computer Science, Applications “G. Parenti”, Florence, Italy; ^4^ Department of Biology, University of Florence, Florence, Italy; ^5^ SOD of Interdisciplinary Internal Medicine, Azienda Ospedaliera Universitaria Careggi (AOUC), Florence, Italy

**Keywords:** cytokines, colorectal cancer, T cells, immune response, gut microbiota

## Abstract

**Background and aim:**

Gut microbiota (GM) can support colorectal cancer (CRC) progression by modulating immune responses through the production of both immunostimulatory and/or immunosuppressive cytokines. The role of IL-9 is paradigmatic because it can either promote tumor progression in hematological malignancies or inhibit tumorigenesis in solid cancers. Therefore, we investigate the microbiota–immunity axis in healthy and tumor mucosa, focusing on the correlation between cytokine profile and GM signature.

**Methods:**

In this observational study, we collected tumor (CRC) and healthy (CRC-S) mucosa samples from 45 CRC patients, who were undergoing surgery in 2018 at the Careggi University Hospital (Florence, Italy). First, we characterized the tissue infiltrating lymphocyte subset profile and the GM composition. Subsequently, we evaluated the CRC and CRC-S molecular inflammatory response and correlated this profile with GM composition, using Dirichlet multinomial regression.

**Results:**

CRC samples displayed higher percentages of Th17, Th2, and Tregs. Moreover, CRC tissues showed significantly higher levels of MIP-1α, IL-1α, IL-1β, IL-2, IP-10, IL-6, IL-8, IL-17A, IFN-γ, TNF-α, MCP-1, P-selectin, and IL-9. Compared to CRC-S, CRC samples also showed significantly higher levels of the following genera: *Fusobacteria*, *Proteobacteria*, *Fusobacterium*, *Ruminococcus2*, and *Ruminococcus*. Finally, the abundance of *Prevotella* spp. in CRC samples negatively correlated with IL-17A and positively with IL-9. On the contrary, *Bacteroides* spp. presence negatively correlated with IL-9.

**Conclusions:**

Our data consolidate antitumor immunity impairment and the presence of a distinct microbiota profile in the tumor microenvironment compared with the healthy mucosa counterpart. Relating the CRC cytokine profile with GM composition, we confirm the presence of bidirectional crosstalk between the immune response and the host’s commensal microorganisms. Indeed, we document, for the first time, that *Prevotella* spp. and *Bacteroides* spp. are, respectively, positively and negatively correlated with IL-9, whose role in CRC development is still under debate.

## Introduction

Colorectal cancer (CRC) is a complex and widespread disease and is the second cause of cancer-related deaths in the world (1). Usually, it begins as benign polyps that can become (especially the adenomatous type) cancerous if not removed: in humans, a variable percentage of polyps, ranging from 1% to 10%, evolve into malignancies (2) because multistep colorectal tumorigenesis does not involve exclusively genetic factors, but also host factors, such as inflammatory and immune responses (3, 4). Indeed, chronic inflammation increases cancer risk through a deregulated activation of the immune system, which causes a loss of tissue architecture and genotoxic cellular DNA damage (5). In this context, CRC is considered the best example of a chronic inflammation–associated tumor, occurring often in patients with inflammatory bowel disease (IBD): IBD-associated CRC is estimated to be 2% of all CRCs, and the rate of death resulting from CRC in IBD patients ranges from 10% to 15% (6, 7). Moreover, according to the immunoediting theory, the adaptive immune system, in addition to protecting the host from developing tumors (8), can support tumor progression. Specifically, T cells can develop different functional features during cancer growth, affecting the disease progression and/or regression. The protective immunity is mediated by effector cells (Th1 and Th17/Th1), and “not effector” T lymphocyte subsets (Th2, Tregs, Tnull) can promote colon cancer progression (9–11). In this scenario, the microbiota plays an important role as well because it is essential to modulate immune responses favoring the equilibrium between protective immunity and tolerance to commensal bacteria (12). A perturbation of the gut microbiota (GM) composition can disrupt this balanced ecosystem, determining a chronic/abnormal immune activation and supporting tumor growth. In fact, over the past 10 years, both specific bacteria and dysbiotic conditions have been associated with or implicated in colorectal carcinogenesis (13, 14) and, in some cases, through the interaction with the immune system (15). In particular, the role of *Fusobacterium nucleatum* is paradigmatic because it promotes CRC by either the induction of epithelial cell proliferation (16), thus generating a proinflammatory microenvironment propitious to cancer progression (17), or *via* the production of proteins able to block the cytotoxic antitumoral activity of T and NK cells (18, 19).

Moreover, microbes can affect cancer cell antigenicity and adjuvanticity (20), determining whether an antigen triggers an immune response and if its nature drives the acquisition of a specific T cell phenotype (effector or regulator). In addition, microbiota can elicit the production of cytokines (and other immune mediators) by influencing the immunostimulatory or immunosuppressive reactions, such as the tendency to mount Th1/Tc1 (characterized by IFN-γ production), Th2/Tc2 (IL-4 and IL-13), Th17/Tc17 (IL-17), or Th9 (IL-9) responses (21–23) that play different roles in colon cancer (11, 24). For example, the commensal bacteria can stimulate the *lamina propria* dendritic cells to produce the IL-6, TGF-β, and IL-23 needed to elicit Th17 and Th9 lymphocyte development (25), and these play a dual role in CRC promotion (5, 26). Current studies show that Th9 cells play a vital antitumor role in most solid tumors (27), but IL-9, as a lymphocyte growth factor, can also promote cancer progression in hematological tumors (23).

Finally, fermentative bacterial products, such as short chain fatty acids, may affect colorectal carcinogenesis by favoring the expression of the Foxp3 gene and boosting Treg functions (28, 29).

Given these premises, our study aims to investigate immune system–microbiota crosstalk in CRC through the cellular and molecular characterization of immunity and the comparative evaluation of microbiota composition in healthy and tumor mucosa, focusing on the correlation between the cytokine profile and GM composition.

## Material and Methods

### Patient Recruitment

In this observational study, 45 patients affected by nonmetastatic colorectal adenocarcinoma at the preoperative stage for undergoing surgical resection were enrolled in 2018 at the Careggi University Hospital (Florence, Italy) (see **Table 1** for a summary of patient characteristics). Exclusion criteria were extraperitoneal rectum localization of the tumor; previous surgery for cancer; previous chemo-radiotherapy treatment; immunodeficiency; travel to exotic areas in the last 5 years; treatment with immunosuppressive drugs, antibiotics, or regular probiotics during the previous 2 months; acute gastrointestinal infections in the month prior to enrollment; and associated presence of established malignancies or chronic intestinal inflammatory diseases (Crohn’s disease and ulcerative recto colitis). Tissue samples of tumor (CRC) and surrounding healthy mucosa (CRC-S) were obtained from the surgical specimen after surgery. The study has received the local ethics committee approval (CE: 11166_spe), and informed written consent has been obtained from each participant.

**Table 1:** CRC patients’ clinical features.

Patient ID	Age	Diagnosis	TNM	Stadium	Site
CRC.1	60-70	Adenocarcinoma	pT2 N0	I	Splenic flexure
CRC.3	70-80	Adenocarcinoma	pT1 N0	I	Sigmoid colon
CRC.4	40-50	Adenocarcinoma	pT3a N1a	IIIB	Sigmoid colon
CRC.5	30-40	Adenocarcinoma	pT3 N0	II	Splenic flexure
CRC.6	80-90	Adenocarcinoma	T1 N0	I	Descending colon
CRC.7	60-70	Adenocarcinoma	pT3 N2b	IIIC	Ascending colon + splenic flexure
CRC.8	70-80	Adenocarcinoma	pT2 N0	I	Hepatic flexure
CRC.9	80-90	Adenocarcinoma	pT2 N0	I	Sigmoid colon
CRC.10	80-90	Adenocarcinoma	pT3 N0	II	Sigmoid colon
CRC.11	70-80	Adenocarcinoma	pT3 N0	II	Sigmoid colon
CRC.13	70-80	Adenocarcinoma	pT3 N0	II	Rectum
CRC.14	60-70	Adenocarcinoma	pT3 N0	II	Sigmoid colon
CRC.15	80-90	Adenocarcinoma	pT3 N1c	IIIB	Rectum
CRC.16	70-80	Adenocarcinoma	pT3 N0	II	Descending colon
CRC.17	80-90	Adenocarcinoma	pT1 N0	I	Sigmoid colon
CRC.18	70-80	Adenocarcinoma	pT2 N1b	IIIA	Cecum
CRC.20	80-90	Adenocarcinoma	pT3 N0	II	Rectum
CRC.22	80-90	Adenocarcinoma	pT3 N1a	IIIB	Rectum
CRC.23	60-70	Adenocarcinoma	T3 N1a G2	IIIB	Splenic flexure
CRC.25	50-60	Adenocarcinoma	pT1 N0	I	Rectum
CRC.28	70-80	Adenocarcinoma	T2 N0	I	Ascending colon
CRC.30	70-80	Adenocarcinoma	pT3 N0	II	Ascending colon
CRC.32	70-80	Adenocarcinoma	T3 N0	II	Rectum
CRC.34	70-80	Adenocarcinoma	pT3 N0	II	Cecum
CRC.36	70-80	Adenocarcinoma	pT3 N0	II	Sigmoid colon
CRC.37	40-50	Adenocarcinoma	pT3 N1b	IIIB	Sigmoid colon
CRC.38	50-60	Adenocarcinoma	T0 N0	NA	Rectum
CRC.39	70-80	Adenocarcinoma	pT3 N0	II	Rectum
CRC.41	60-70	Adenocarcinoma	pT1 N0	I	Rectum
CRC.44	80-90	Adenocarcinoma	pT3 N0	II	Ascending colon
CRC.46	70-80	Adenocarcinoma	pT3 N1c	IIIB	Rectum
CRC.47	60-70	Adenocarcinoma	pT2 N0	I	Cecum
CRC.48	40-50	Adenocarcinoma	pT3 N0	II	Cecum
CRC.50	60-70	Adenocarcinoma	pT3 N0	II	Recto-sigmoid junction
CRC.51	80-90	Adenocarcinoma	pT3 N0	II	Descending colon
CRC.52	60-70	Adenocarcinoma	pT3 N0	II	Hepatic flexure
CRC.54	70-80	Adenocarcinoma	pT2 N0	I	Hepatic flexure
CRC.55	70-80	Adenocarcinoma	pT3 N0	II	Rectum
CRC.56	60-70	Adenocarcinoma	pT3 N0	II	Ascending colon
CRC.59	70-80	Adenocarcinoma	pT3 N1b	IIIB	Recto-sigmoid junction
CRC.65	70-80	Adenocarcinoma	pT2 N1c	IIIA	Rectum
CRC.71	80-90	Adenocarcinoma	pT2 N	I	Ascending colon
CRC.73	50-60	Adenocarcinoma	T3 N0	II	Sigmoid colon
CRC.75	70-80	Adenocarcinoma	T4b N0	II	Transverse colon
CRC.76	70-80	Adenocarcinoma	pT1 N0	I	Recto-sigmoid junction

### Immunological Analysis

#### Analysis of Tissue Infiltrating Lymphocytes (TILs)

Tissue samples were dissociated with the Tumor Dissociation Kit, human (Miltenyi Biotech, UK) in combination with the gentleMACS™ Octo Dissociator (Miltenyi Biotech, GmbH) to obtain a gentle and rapid generation of single-cell suspensions. Then, TILs were magnetically isolated with antihuman CD3 microbeads (Miltenyi Biotech, UK) using the AutoMACS Pro Separator (Miltenyi Biotech, GmbH) and analyzed by polychromatic flow cytometry. In detail, TILs from dissociated tissues were characterized for the expression of CD4, CD8, CD25, CD127, IFN-γ, IL-4, IL-17, IL-9, IL-22, and FoxP3 using intracellular cytokine staining. Briefly, TILs were cultured in RPMI 1640 culture medium (SERO-Med GmbH, Wien) supplemented with 10% FCS HyClone (Gibco Laboratories, Grand Island, NY, USA) and stimulated for 5 h using the Leukocyte Activation Cocktail with BD GolgiPlug™ (BD Pharmingen). Cells were stained for surface antigens and then fixed with 4% (v/v) paraformaldehyde and permeabilized with 0.5% saponin, followed by intracellular staining with anti-IL-4, anti-IL-17, anti-IL-22, anti-IL-9, and anti-IFN-γ mAbs (BD Biosciences). For the detection of peripheral Tregs, TILs were fixed and permeabilized using the BD Pharmingen Human FoxP3 Buffer Set (BD Biosciences). A minimum of 10,000 events were acquired.

#### Molecular Inflammatory Response Evaluation

In a restricted court of patients (*n*=14) for whom the tissue was available, we evaluated the tumor and healthy mucosa–associated inflammatory response through the evaluation of 26 cytokines by specifically assembled MixMatch Human kits for Luminex MAGPIX detection system (Affymetrix, eBioscience) and following the manufacturers’ instructions. More specifically, we analyzed macrophage inflammatory protein-1α (MIP-1α), interleukin (IL)-27, IL-1β, IL-2, IL-4, IL-5, interferon gamma-induced protein 10 (IP-10), IL-6, IL-8, IL-10, IL-12p70, IL-13, IL-17A, granulocyte-macrophage colony stimulating factor (GM-CSF), tumor necrosis factor-α (TNF-α), interferon (IFN)-α, IFN-γ, monocyte chemotactic protein 1(MCP-1), IL-9, P-selectin, IL-1α, IL-23, IL-18, IL-21, soluble intercellular adhesion molecule-1 (sICAM-1), and IL-22. The levels of cytokines were estimated using a 5-parameter polynomial curve (ProcartaPlex Analyst 1.0).

The low and upper limit of quantification (LLOQ and ULOQ) used for the cytokines and chemokines are reported in **Table 2**. A value under the LLOQ was considered to be 0 pg/ml.

**Table 2:** Low and Upper Limit of Quantification (LLOQ and ULOQ) for each evaluated cytokine/chemokine.

Cytokine/chemokine	ULOQ (pg/ml)	LLOQ (pg/ml)	
**MIP-1α**	1880	1,84
**IL-27**	82000	20
**IL-1β**	8250	2,01
**IL-2**	26900	6,57
**IL-5**	30400	7,42
**IP-10**	7750	1,89
**IL-6**	37800	9,23
**IL-8**	9850	2,4
**IL-17A**	9550	2,33
**IFN- γ**	12675	12
**GM-CSF**	41000	10
**TNF-α**	29500	7,2
**MCP-1**	14800	3,61
**IL-9**	31000	7,57
**IL-1α**	3000	0,73
**IL-18**	49500	12
**IL-21**	39700	9,69
**IL-22**	82500	20
**P-selectin**	5051600	1233
**sICAM1**	870200	212
**IL-4**	36200	8,84
**IL-10**	9250	2,26
**IL-12p70**	28100	6,86
**IL-13**	13400	3,27
**IL-23**	68500	17
**IFN-α**	2250	0,55

MIP, Macrophage Inflammatory Proteins; IL, Interleukin; IP, Interferon gamma-induced protein; IFN, Interferon; GM-CSF, Granulocyte-Macrophage Colony-Stimulating Factor; TNF, Tumor necrosis factor; MCP, Monocyte Chemoattractant Protein; sICAM1, soluble Intercellular Adhesion Molecule.

#### Statistical Analysis of Immunologic Data

Statistical analysis was performed with SPSS statistical software (version 24). Differences between T cells subset data obtained from CRC and CRC-S samples and tissue cytokine levels evaluated in the same groups were assessed with paired Wilcoxon signed-rank tests. *P* values less than 0.05 were considered statistically significant.

### Microbiota Characterization

#### DNA Extraction

Genomic DNA was extracted using the DNeasy PowerLyzer PowerSoil Kit (Qiagen, Hilden, Germany) from frozen (-80°C) CRC and CRC-S according to the manufacturer’s instructions. Briefly, tissues were added to a bead beating tube and thoroughly homogenized with TissueLyser II for 5 min at 30 Hz. Total genomic DNA was captured on a silica membrane in a spin column format and subsequently washed and eluted. The quality and quantity of extracted DNA was assessed using the NanoDrop ND-1000 (Thermo Fisher Scientific, Waltham, US) and the Qubit Fluorometer (Thermo Fisher Scientific), respectively. Then, genomic DNA was frozen at -20°C.

#### Bioinformatic Analysis of 16S rRNA

Extracted DNA samples were sent to IGA Technology Services (Udine, Italy), where amplicons of the variable V3–V4 region of the bacterial 16s rRNA gene were sequenced in paired-end (2 × 300) cycles on the Illumina MiSeq platform, according to the Illumina 16S Metagenomic Sequencing Library Preparation protocol (30).

Raw sequences were processed following the software pipeline MICCA (31). Paired-end reads were assembled using the “mergepairs” command, maintaining a minimum overlap of 100 bp and an edit distance in the maximum overlap of 32 bp. Subsequently, the sequences were cut with the “trim” command to remove the primers and eventually eliminate the reads with imperfect primer sequences. All the reads with a length lower than 350 bp and with an error rate higher than or equal to 0.5 were removed with the “filter” command.

Clean reads were eventually merged into a single file with the “merge” command and transformed into a FASTA format file. The operational taxonomic units (OTUs) were generated using the “otu” command in “denovo_greedy” mode, setting a 97% identity and performing an automatic removal of chimeras with the “-c” option. The longest sequence of each OTU was used for taxonomic assignment using the “classify” command in “rdp” mode, i.e., using the RDP Bayesian classifier that is able to obtain classification and confidence for taxonomic ranks up to genus.

#### Statistical Analysis of Bacterial Communities

Statistical analyses on the bacterial communities were performed in R (R Core Team, 2014) with the help of the packages phyloseq 1.26.1 (32), DESeq2 1.22.2 (33), breakaway 4.6.16 (34, 35), and other packages satisfying their dependencies—in particular, vegan 2.5-5 (36). Rarefaction analysis on OTUs was performed using the function rarecurve (step 50 reads) and further processed to highlight saturated samples (arbitrarily defined as saturated samples with a final slope in the rarefaction curve with an increment in OTU number per reads < 1e-5).

For the cluster analysis (complete clustering on Euclidean distance) of the entire community, the OTU table was first normalized using the total OTU counts of each sample and then adjusted using square root transformation.

The coverage was calculated by Good’s estimator (37) using the formula: (1 - *n*/*N*) × 100, where *n* is the number of sequences found once in a sample (singletons) and *N* is the total number of sequences in that sample.

Richness, Shannon, Chao 1, and Evenness indices were used to estimate bacterial diversity in each sample using the function estimate_richness from phyloseq (31). The evenness index (38) was calculated using the formula *E* = *S*/log(*R*), where S is the Shannon diversity index and R is the number of OTUs in the sample. Differences in all indices between CRC and CRC-S were tested using a paired Wilcoxon signed-rank test. Sample richness was further measured using the estimator and its associated error introduced in the breakaway package (32). The function betta_random of the breakaway package was further used to evaluate the statistical differences in richness between paired-by-patient CRC and CRC-S samples.

The differential analysis of abundance was performed with DESeq2 (31) at the OTUs and at the different taxonomic ranks (created using the tax_glom function in phyloseq) by using a two-group blocked-by-patient design to perform a paired test.

### Statistical Analysis of the Association Between Tissue Microbiota and Cytokines

The association between tissue microbiota and cytokines was investigated with a 2-step analysis separately for the mucosa and tumor tissues. In the first step, we implemented a modified version of the sure independence screening (SIS) procedure (39). SIS uses the notion of marginal correlation—in our case, the correlation of a single cytokine with the dependent variable—to rank the cytokines. The cytokines with the smallest *p*-value from a Dirichlet regression (40) with that given cytokine as the only predictor are included in step 2. For each cytokine, the *p*-value is obtained testing the model with the considered cytokine and the intercept against a model with only the intercept with a likelihood-ratio test. Step 1 is necessary only when the list of cytokines is too long compared with the sample size; in our analysis, we selected the three most relevant cytokines from step 1.

In the second step, we used Dirichlet multinomial regression to determine the joint effect of cytokines on the tissue microbiota. We implemented a Bayesian variable selection (BVS) method based on a thresholding function (41). This approach is based on a Monte Carlo Markov chain algorithm that explores the space of possible models. The method’s output is a list of posterior probability of inclusion (PPI) and the posterior mean of the nonzero regression coefficients. PPI is the probability, between 0 and 1, that a given association cytokine-genera is nonzero, accounting for the effect of all other cytokines. The posterior mean is an estimate of a nonzero association. Each estimated regression coefficient evaluates the taxon–cytokine association, whose sign and magnitude measure the effect of the cytokine on the taxon.

## Results

### Assessment of Tissue Infiltrating T Cell Subset Distribution in Healthy and Cancer Mucosa

We performed polychromatic flow cytometry analysis of TILs isolated from the dissociated CRC and CRC-S. The percentage of CD4^+^ and CD8^+^ TILs in the mucosa sample group did not differ significantly. In detail, the mean percentages (SD) of CD4^+^ cells were 58.03 (6.88) in CRC vs. 58.15 (6.32) in CRC-S and the mean percentages of CD8^+^ T cells were 17.42 (5.65) in CRC vs. 14.39 (4.14) in CRC-S.

**Figure 1:**
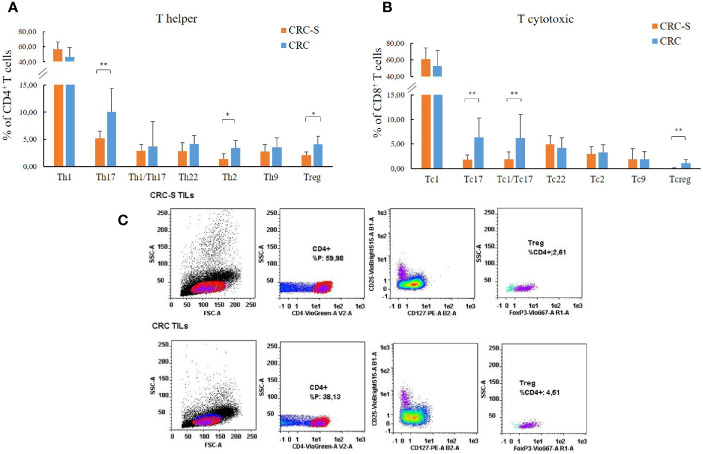
T cell subset distribution in the tumor mucosa and surrounding healthy mucosa samples groups. Panel **(A)** reports the mean percentages (+SD) of T helper subsets with respect to the percentage of CD4^+^ T cells, and panel **(B)** reports the mean percentages (+SD) of T cytotoxic subsets with respect to the percentage of CD8^+^ T cells. Panel **(C)** reports the cytofluorimetric analysis of T regulatory cells in the tumor mucosa and surrounding healthy mucosa samples of one representative patient. Statistical analyses were calculated using Wilcoxon signed-rank test. The asterisks (*) represent *p*-values, *p < 0.01, **p < 0.001. CRC = tumor mucosa; CRC-S = healthy mucosa.

The analysis of the T cell subsets revealed that the tumor mucosa sample group displayed higher percentages of Th17 (CRC vs. CRC-S: 10.02 (4.32) vs. 5.13 (1.39); *p*=0.0008), Th2 (CRC vs. CRC-S: 3.45 (1.31) vs. 1.41 (0.93); *p*=0.0011), and Treg (CRC vs. CRC-S: 4.08 (1.44) vs. 2.10 (0.57); *p*=0.0040) as shown in **Figures 1A, C**. Regarding the T cytotoxic cells, the CRC group showed higher percentages of Tc17 (CRC vs. CRC-S: 6.33 (3.98) vs. 1.77 (1.00), *p*=0.0036), Tc1/Tc17 (CRC vs. CRC-S: 6.25 (4.74) vs. 1.88 (1.48), *p*=0.0022), and Tcreg (CRC vs. CRC-S: 1.08 (0.81) vs. 0.06 (0.08), *p*=0.0055) (**Figure 1B**). Notably, the number of Th9s is major (but not significant) in CRC tissue, and the Tc9s are similar in the two different sites.

### Molecular Inflammatory Profile in CRC-Associated Tissues

**Figure 2:**
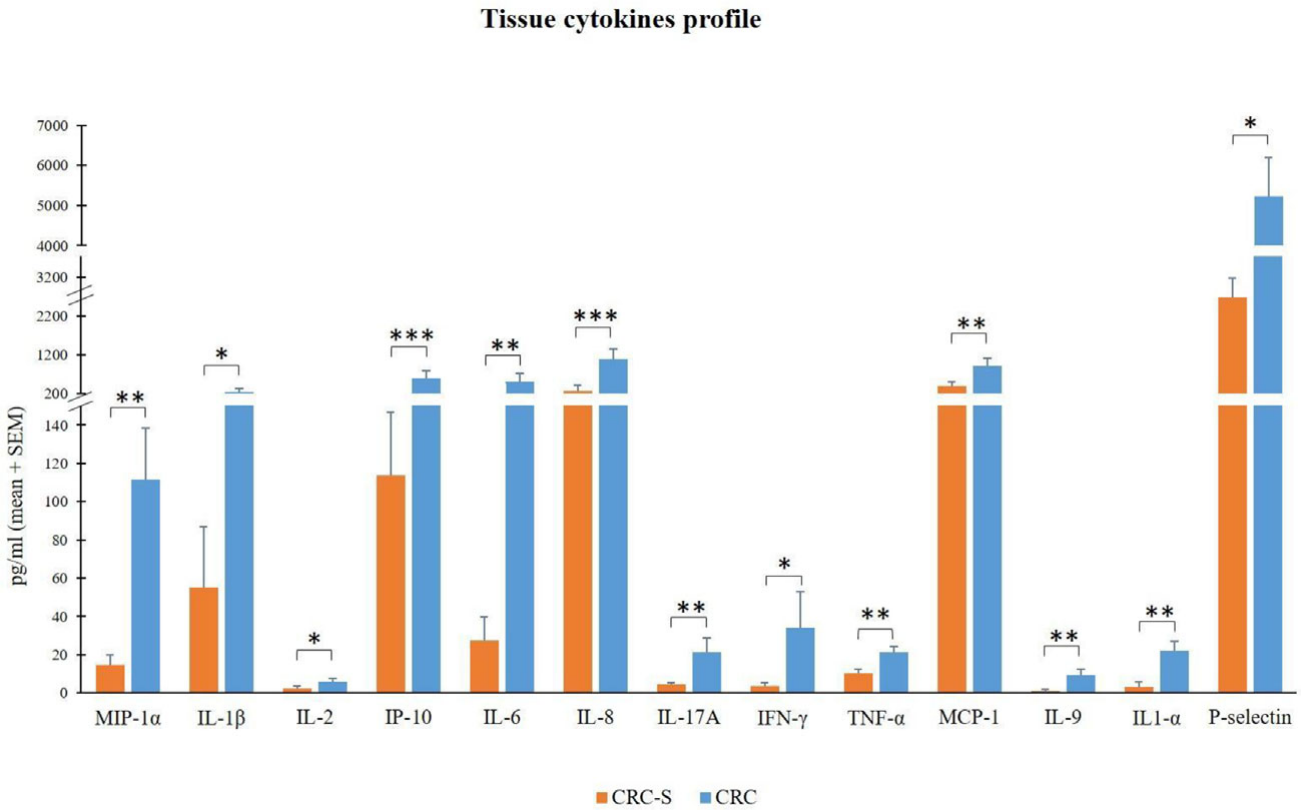
Tissue cytokine levels in 14 CRC patients. The histogram reports the mean (+SEM) cytokine levels (pg/ml) of the evaluated cytokines in CRC-S and CRC of 14 CRC patients. Wilcoxon signed-rank test was performed to test the differences between CRC-S and CRC paired samples. A *p*-value < 0.05 is considered statistically significant. The asterisks (*) represent *p*-values, *p < 0.05, **p < 0.01, ***p < 0.001. CRC-S= healthy mucosa; CRC= tumor mucosa.

We compared the molecular inflammatory profile of the homogenized CRC and CRC-S of 14 cancer patients through the evaluation of 26 pro- and anti-inflammatory cytokines. Six of the evaluated cytokines (IL-4, IL-10, IL12p70, IL-13, IL-23, and IFN-α) were under the LLOQ (**Table 2**) in all samples either because levels were very low (not detectable) or these molecules are not produced. The other 20 cytokines showed a common trend in all patients, characterized by higher levels in CRC compared to CRC-S. In particular, IL-1β, IL-2, IFN-γ, P-selectin, MIP-1α, IL-6, IL-17A, TNF-α, MCP-1, IL-9, IL-1α, IP-10, and IL-8 were increased significantly in CRC compared to CRC-S (**Figure 2**), whereas IL-27, IL-21, IL-22, IL-18, IL-5, GM-CSF, and sICAM1 showed a similar but not significant trend (*p* > 0,05).

### Comparison of Mucosal Microbiota Composition in CRC and CRC-S

Our sequencing efforts in assessing microbiota composition encompassed a total of 12,475,251 reads for 40 sample pairs. After all the preprocessing steps, which included pair merging, trimming, quality filtering, and chimera detection, a total of 8,458,126 (67.8%) were available for further analysis.

**Figure 3:**
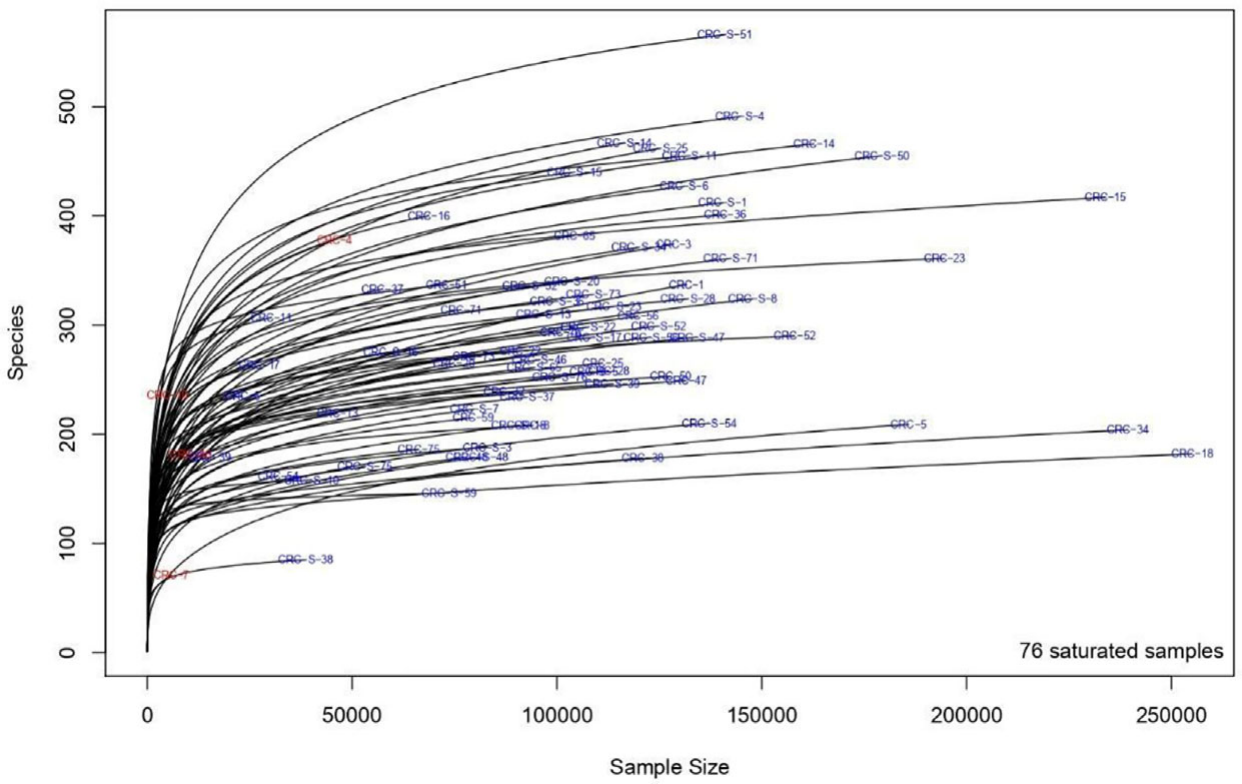
Rarefaction curves showing the level of saturation of OTUs.

Saturation curves (**Figure 3**) revealed that most specimens were sufficiently sampled. Samples showed a Good’s coverage ranging from 99% to 100%, indicating that less than 1% of the reads in a given sample came from OTUs that appeared only once in that sample.

**Figure 4:**
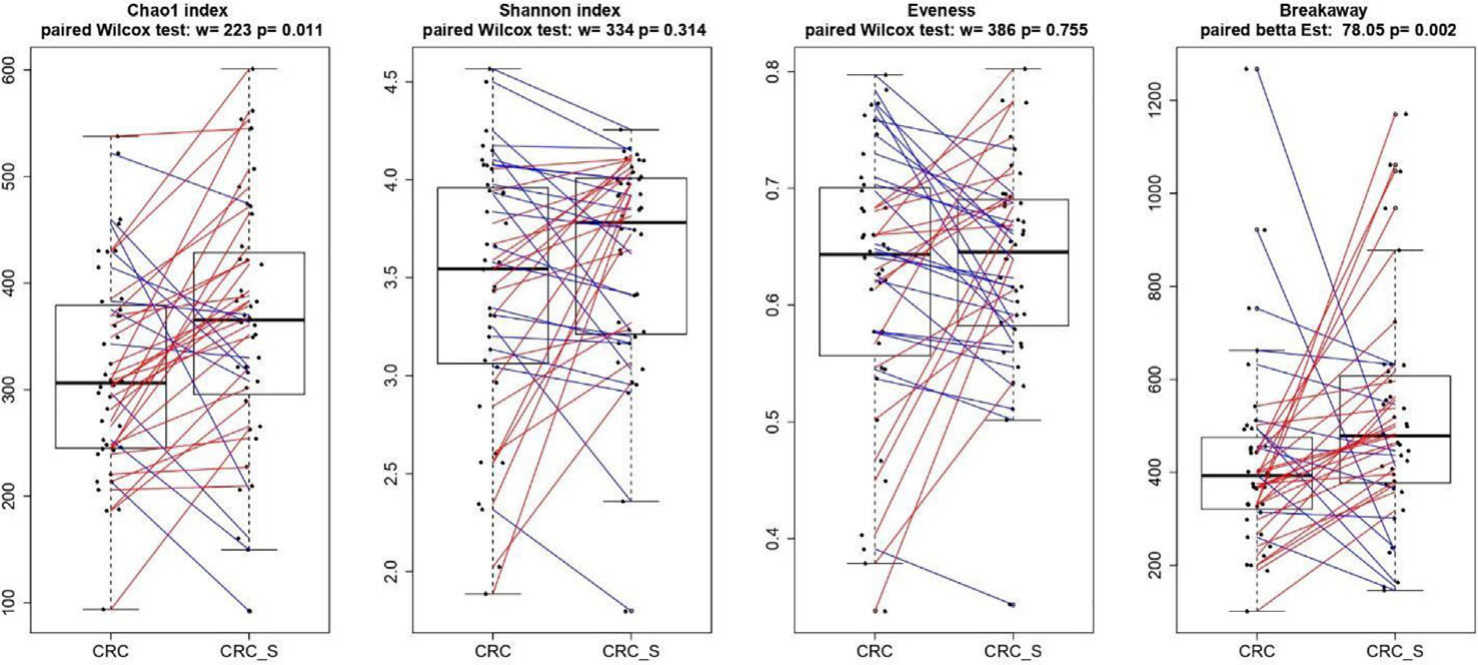
Boxplots showcasing alpha diversity indices (Chao1 index, Shannon index, Evenness, and Breakaway) in CRC and CRC-S samples. Statistical differences were evaluated using paired Wilcoxon signed-rank test for Chao, Shannon, and Evenness indices and using the paired betta analysis implemented in the Breakaway R package. *P*-values less than 0.05 were considered statistically significant.

As shown in **Figure 4**, the alpha diversity of samples did not display significant differences for Shannon index and Evenness. On the contrary, a significant (*p* = 0.011) Chao1 index evidenced that rare OTUs are enriched in CRC-S vs. CRC, denoting a higher diversity.

Taxonomic analysis detailed in **Table 3** reveals for the 2454 OTUs formed the confident (<20% error) presence of 29 phyla (>99% reads), 50 classes (>98% reads), 87 orders (>98% of reads), 176 families (>96% reads), and 372 genera (>86% reads).

**Table 3:** Summary of the taxonomic analysis of the obtained OTUs.

Rank	Count	Reads	Reads.	OTU	OTU%
Phylum	29	8205619	99.58	2325	94.74
Class	50	8146401	98.86	2234	91.04
Order	87	8114962	98.48	2177	88.71
Family	176	7919409	96.11	1934	78.81
Genus	372	7107700	86.26	1290	52.57

**Figure 5:**
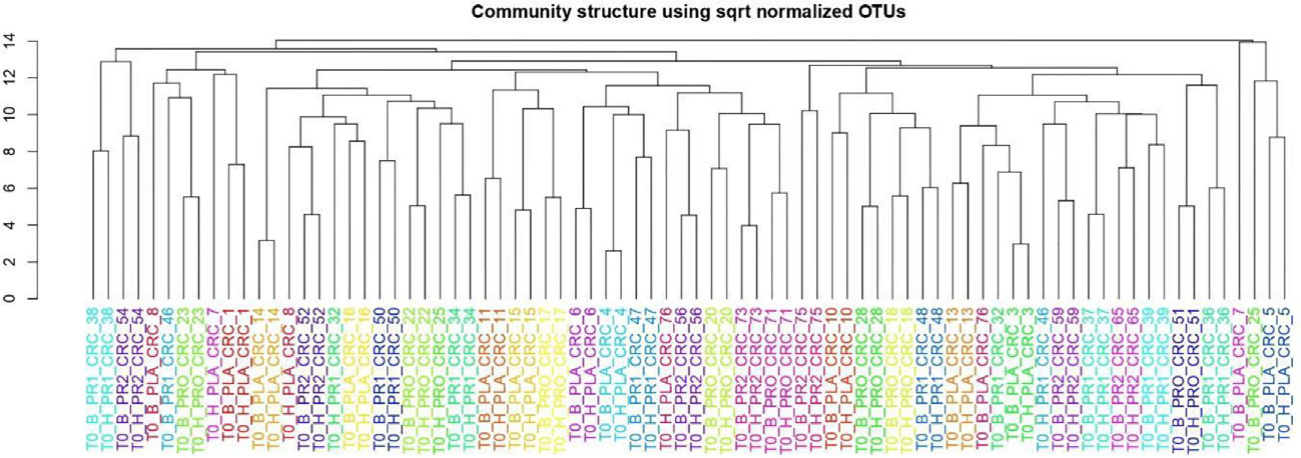
Cluster analysis on normalized OTU counts.

**Figure 6:**
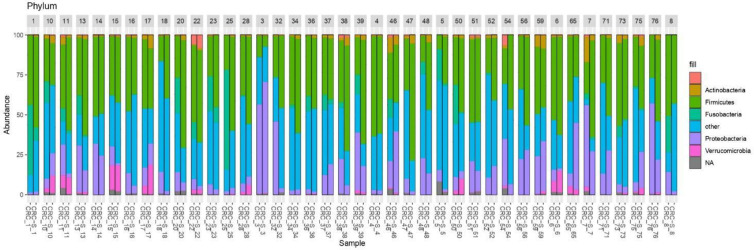
Stacked boxplots of microbial composition at phylum level of CRC and CRC-S samples.

To investigate and confirm the paired nature of sampling (i.e., tumor tissue vs. surrounding healthy tissue), we performed a cluster analysis on normalized OTU counts. As shown in **Figure 5**, we verified that 36/40 paired samples were, in effect, also paired in terms of microbial composition, a result robust to changes in distance metrics (e.g., Bray-Curtis) and the clustering method (data not shown). The analysis of the taxonomic composition reported that 6 phyla dominated the data set (98% sequences), namely *Firmicutes* (41.24%), *Bacteroidetes* (35.89%), *Proteobacteria* (13.37%), *Fusobacteria* (4.68%), *Verrucomicrobia* (2.10%), and *Actinobacteria* (1.56%) as shown in **Figure 6**. Stacked boxplots of taxa abundance at different taxonomic ranks are available as (**Supplementary Figures S1**–**S4**).

The paired comparison of the abundance of single OTUs revealed significant (adj. *p*<0.05, abs (logFC)>=1) differences between CRC and CRC-S sample groups with 6.6% OTUs involved. At the phylum level, *Fusobacteria* and *Proteobacteria* were significantly higher in CRC compared to CRC-S (logFC = -2.92, adj. *p* = 6.17e-15 and logFC = -0.95, adj. *p* = 1.92e-05, respectively). At the genus level, 14.7% genera were observed as significantly (adj. *p*<0.05, abs (logFC)>=1) different, the most abundant being *Fusobacterium* (average OTUs 6199, log2FC = -2.93, adj. *p* = 1.06e-08), *Ruminococcus2* (*Lachnospiraceae* family, average OTUs 2911, log2FC = 1.31, adj. *p* = 1.38e-3), and *Ruminococcus* (*Ruminococcaceae* family, average OTUs 1640, log2FC = 1.82, adj. *p* = 4.12e-05).

### Correlation of the Cytokine Profile With the Microbiota Composition in CRC-Associated Tissues

To evaluate this critical and crucial point, we first applied the SIS screening procedure (step 1). We considered the OTU counts of the three most abundant genera and aggregated all other genera in a residual category. Our dependent variable was then defined by four categories: *Bacteroides* spp., *Prevotella* spp., and *Escherichia/Shigella* spp. (plus the residual category). The four top-ranked cytokines were IL-18, IFN-γ, IL-5, and IL-2. Using these four cytokines (plus the intercept), we ran the BVS method (step 2). In the tumor tissues, we detected the association between IL-5 and *Prevotella* spp. with a PPI=0.81; the same association was found to be supported by the data collected from the mucosa tissues as well (PPI=0.77). The effect of IL-5 on *Prevotella* is estimated to be positive and equal to 0.64 (posterior mean) and 0.86 for tumor and mucosa tissues, respectively. However, this analysis hardly describes the entire picture because many cytokines with very small *p*-values in the first step were not included in the second step due to computational constraints.

Moreover, we decided to perform a second analysis that includes directly into step 2 the cytokines that showed differential abundances in comparative analysis between CRC and CRC-S samples. However, due to the computational constraints that impose a limit on the sample size, we selected some of the cytokines that were significantly increased in CRC compared to CRC-S samples, according to their relevance in the current literature. In particular, we chose IFN-γ, IL-17A, IL-8, IL-1β, IL-1α, IP-10, MIP-1α, and IL-9. The results of the BVS Dirichlet multinomial regression (step 2) are reported in **Tables 4** and **5**. From the results, we notice that *Prevotella* spp. is associated with both IL-17A and IL-9. The first association is negative, and the effect is 1.08 (posterior mean), whereas the second is positive, with a posterior mean of 1.37. *Bacteroides* spp. and *Escherichia/Shigella* spp. show a negative association with IL-9 and IP-10, respectively, with posterior means equal to -0.91 and -0.89, respectively.

**Table 4:** Posterior probabilities of inclusion (PPIs).

	*Prevotella* spp.	*Bacteroides* spp.	*Escherichia/Shigellaspp.*	Residual category
**intercept**	1.00	1.00	1.00	1.00
**IFN-γ**	0.10	0.00	0.45	0.00
**IL-17A**	0.82	0.00	0.00	0.00
**IL-8**	0.00	0.00	0.00	0.00
**IL-1β**	0.00	0.00	0.00	0.00
**IL-1α**	0.00	0.00	0.00	0.00
**MIP-1α**	0.00	0.00	0.00	0.00
**IP-10**	0.00	0.00	0.77	0.00
**IL-9**	0.95	0.83	0.00	0.00

**Table 5:** Posterior mean of the regression coefficients.

	*Prevotella* spp.	*Bacteroides* spp.	*Escherichia/Shigellaspp.*	Residual category
intercept	-1.10	0.41	-0.93	1.90
IFN-γ	-0.05	0.00	0.31	0.00
IL-17A	-1.08	0.00	0.00	0.00
IL-8	0.00	0.00	0.00	0.00
IL-1β	0.00	0.00	0.00	0.00
IL-1α	0.00	0.00	0.00	0.00
MIP-1α	0.00	0.00	0.00	0.00
IP-10	0.00	0.00	-0.89	0.00
IL-9	1.37	-0.91	0.00	0.00

## Discussion

In this study, we first explored the immunity–microbiota axis in human CRC, comparing the distribution of TILs, the cytokine profile, and the GM composition in cancerous and surrounding mucosa. In agreement with our previous findings (11), the TIL assessment revealed higher percentages of tumor-supporting T cell subsets (Th17, Th2, Th9, and Tregs) in CRC samples compared to CRC-S. Contextually, a Th2 shift in the tumor microenvironment, especially for CRC, strongly contributes to cancer relapse, metastasis, and worse prognosis (42, 43) although, according to existing contradictory evidence, excess inflammation caused by CD4^+^ and CD8^+^ IL-17-producing T cells or the immunosuppression induced by Tregs may lead to carcinogenesis (44, 45). Indeed, tumor-infiltrating Th17 and Tc17 cells have been found in various human cancers, confirming their protumorigenic properties (46, 47). According to our results, different studies found higher percentages of Th17 and Tc1/Tc17 cells in tumor tissues compared to adjacent nontumor tissues (48–50). Through their immunosuppressive properties, Tregs can favor immune escape mechanisms of tumor cells, and that is why high amounts of peripheral or tumor-infiltrating Tregs are often associated with poor clinical outcome in gastrointestinal cancer (51, 52). According to our findings, a high number of tumor-infiltrating Tregs is widely documented (53–55).

Finally, Th9 cells, a relatively new subset, seem to have a dual role in tumor progression. Generally, the Th9 cells (activating both the innate and adaptive immune responses) and the Tc9s play an important role in antitumor immunity (56, 57), but IL-9 can exert a tumorigenic role in both hematological and solid tumors (58).

In addition to the TIL analysis, although many studies investigate the levels of several plasma cytokines in CRC patients (59–62), we assessed for the first time the molecular inflammatory profile of CRC mucosa through the evaluation of an exhaustive panel of 26 cytokines of which 20 were increased in CRC compared to CRC-S. In particular, CRC samples revealed significantly higher levels of MIP-1α, IL-1β, IL-2, IP-10, IL-6, IL-8, IL-17A, IFN-γ, TNF-α, MCP-1, IL-1α, P-selectin, and IL-9.

The relevant higher percentages of chemokines MCP-1, MIP-1α, IL-8, and IP-10 reflect the high colonic inflammation, and many studies demonstrate their role in the development of a tumor-favoring microenvironment due to their abilities to favor angiogenesis and to stimulate macrophages and CD8^+^ T cell recruitment *in situ* (63–65). In accordance with our results, different studies found high levels of these chemokines in CRC tissues (66–68).

In addition, the cytokines IL-1α, IL-1β, IL-6, IL-17A, and TNF-α promote tumor initiation, progression, angiogenesis, and metastasis in many human malignancies, including CRC (69–71), and our findings are consistent with previous studies (72–75).

Finally, although the overexpression of IFN-γ in CRC tissues can be considered positive (for its established robust antitumor activity) (76, 77), IL-2 and IL-9 display both pro- and antitumor potentials (78, 79). About IL-9, in contrast with our results, Wang et al. show that IL-9 is less expressed in human colon carcinoma (80). Nevertheless, Huang et al. report low IL-9 amounts in CRC patients, but these low levels are associated with tumor progression (81). Interestingly, Tian and colleagues show that IL-9 expression in colitis-associated cancer tissue is significantly higher than that in adjacent tissues, and Lentiviral vector–mediated IL-9 overexpression in the colon cancer cells lines RKO and Caco2 could promote their proliferation (82).

The relationship between CRC development and GM imbalance has been well established in past years (83–85), and numerous studies document distinct fecal and mucosal microbiota profiles in CRC patients compared with healthy subjects (86–88). Indeed, CRC-associated microbiota profiles differ from those in healthy subjects (87), and a brilliant meta-analysis of eight geographically and technically diverse fecal shotgun metagenomic studies of CRC identifies a peculiar colon cancer signature (88). Hence, we characterized the microbiota composition of tumor and adjacent healthy mucosa in the enrolled CRC patients, and according to previous findings, a significant Chao1 index evidences that rare OTUs are enriched in CRC-S vs. CRC (89, 90). We also confirm our recent data (86), demonstrating that Fusobacteria and *Fusobacterium* spp. are associated with CRC and are amplified during colorectal carcinogenesis (91–93), and we find a significantly higher percentage of Proteobacteria in CRC, according to evidence that an imbalanced GM is often associated with a sustained increase in Proteobacteria phylum members (94, 95). Consistent with Weir et al (96), we find that *Ruminococcus* spp. are more represented in CRC even if some authors report that, in CRC patients, *Ruminococcus* spp. have low prevalence (97, 98).

Moreover, to explore the mucosal microbiota–local immune response axis, we correlate—for the first time—the cytokine profile and GM composition using BVS Dirichlet multinomial regression. The application of the SIS screening procedure and the OTU counts of the three most abundant genera (*Bacteroides* spp., *Prevotella* spp., *Escherichia/Shigella* spp., and all other genera aggregated in a residual category), allowed us to identify the four top-ranked cytokines (IL-18, IFN-γ, IL-5, and IL-2) that we used to run the BVS method. Although these analyses cannot adequately describe the complex scenario of the relationship between secreted cytokines and intestinal composition, we observed a positive association between IL-5 and *Prevotella* spp. in both tumor and mucosa tissues.

As is well known, IL-5 is essential for eosinophil differentiation, and eosinophilia has been observed in various cancers, including CRC, with a controversial prognosis link. Eosinophil infiltration is considered unfavorable in Hodgkin’s lymphoma but positive in breast and prostate cancers (99). As recently reviewed (100), higher numbers of infiltrating eosinophils detected in CRC tissue were repeatedly shown to be prognostically favorable (101–103), but the mechanisms of CRC growth inhibition remain poorly understood. A recently developed CRC mouse model shows that tumor-homing eosinophils secrete chemoattractants for CD8^+^ effector T cells, eventually causing tumor rejection (104).

Furthermore, abundant IL-5 levels are documented in the synovium of rheumatoid arthritis patients (105), and notably, Scher et al. show that the presence of *Prevotella copri* is strongly correlated to rheumatoid arthritis (106). Therefore, taking into account all these reported data, our results—showing a positive correlation between IL-5 and *Prevotella* spp.*—*and the IL-5 anti-inflammatory role, we can assume an attempt to restore a eubiotic ecosystem in the colon mucosa contrasting the CRC development.

In the BVS method, we include all cytokines that show differential abundances in the comparative analysis of CRC and CRC-S: IFN-γ, IL-17A, IL-8, IL-1β, IL-1α, IP-10, MIP-1α, and IL-9. For the first time, we find that, again, *Prevotella* spp. is negatively associated with IL-17A but positively related to IL-9. In addition, *Bacteroides* spp. and *Escherichia/Shigella* spp. show a negative association with IL-9 and IP-10, respectively.

Despite the negative correlation between *Prevotella* spp. and IL-17A, it has recently been discovered that Prevotellaceae are able to promote Th17 cell differentiation, and *Prevotella* spp. are associated with Th17-mediated diseases, including periodontitis and rheumatoid arthritis (107). These conflicting data could be explained by speculating that the same bacterial taxa, in different environmental conditions, may exert contrasting effects. However, we cannot exclude that contrasting results may derive from the relatively low level of taxonomic resolution of 16S rRNA gene metagenomics, which cannot fully discriminate between different species of the same genus (as *Prevotella* spp.). Indeed, different *Prevotella* species colonize the human body districts. The periodontal *Prevotella* spp. related to Th17 cell stimulation could not be subdued to a dysbiotic environment, such as the inflamed CRC mucosa. In addition, in a study in which *Prevotella histicola* was used to modulate immune response and treat arthritis in a humanized mouse model, Marietta et al. report that treated mice showed significantly lower levels of IL-17 in agreement with our data. The authors also report decreased level of IL-9 as compared to placebo-treated mice in contrast with our results (108). Finally, Campisciano (109) detected an increase of the relative vaginal abundance of *Prevotella timonensis* in women infected with HPV, which showed a decreased concentration of the IL-15, IL-7, and IL-9 that they associated with the virus infection.

Regarding the negative correlation between *Bacteroides* spp. and IL-9, we have not found studies documenting this association, but because it is well known that IL-9 is produced by Th17, we have indirect evidence of this correlation. Round et al. find that *Bacteroides fragilis* inhibits Th17 development, inducing Treg accumulation, and Vaahtovuo et al. demonstrate the lower abundance of *Bacteroides* spp. in rheumatoid arthritis patients (110, 111).

Our results also show that *Escherichia/Shigella* spp. are negatively correlated with the IP-10 that is induced in many viral, bacterial, and parasite infections, i.e., shigellosis and *E. coli* infection (112, 113). Because IP-10 is increased in CRC samples, the negative correlation between *Escherichia/Shigella* spp. also can be supported.

In conclusion, our data describe a clear dissimilarity of the cellular and molecular inflammatory profile and intestinal microbiota composition between the tumor and the adjacent healthy tissue, displaying the generation of a peculiar CRC microenvironment. The infiltrating T cell features and the higher percentages of several cytokines produced in the tumor tissue document that, among all different types of immune cells involved in the complex anticancer responses, some may even encourage neoplastic progression. In addition, the distinct microbiota CRC profile may suggest that microbial communities can drive and modulate the antitumor immune response. In fact, we show—for the first time in human CRC—that *Prevotella* and *Bacteroides* species are correlated positively and negatively, respectively, with the IL-9 that has an intriguing and still debated role in tumor immunity. We are aware that other studies in humans and in animal models are needed, but the observed correlation of the cytokine signature with the GM composition confirm the presence of bidirectional crosstalk between the immune response and the host’s commensal microorganisms, which may influence cancer development.

## Data Availability Statement

The datasets presented in this study can be found in online repositories. The names of the repository/repositories and accession number(s) can be found in the article/**Supplementary Material**.

## Ethics Statement

The studies involving human participants were reviewed and approved by Comitato Etico Area Vasta Toscana Centro. The patients/participants provided their written informed consent to participate in this study.

## Author Contributions

EN, ER, SB, and AA conceived and designed the study, and drafted the paper. EN, FR, and GN acquired experimental data of immune response. ER, AM, RF, GB, CF, and CC acquired experimental data of microbiota. AT and MNR were involved in enrolment and obtaining clinical data of patients. MP and FS are responsible for the statistical analysis. MR is responsible for bioinformatics’ analysis of microbiota. EN, SB, FS, MR, and AA analyzed and interpreted the data. AA, PB, and DP critically revised the paper. All authors contributed to the article and approved the submitted version.

## Conflict of Interest

The authors declare that the research was conducted in the absence of any commercial or financial relationships that could be construed as a potential conflict of interest.
