# Supplementary material for: Significant and Conflicting Correlation of IL-9 With Prevotella and Bacteroides in Human Colorectal Cancer
Source: Front Immunol. 2021 Jan 8;11:573158. doi: 10.3389/fimmu.2020.573158 (PMC7820867; doi:10.3389/fimmu.2020.573158)
Supplement: Supplementary file 1 [file DataSheet_1.pdf]

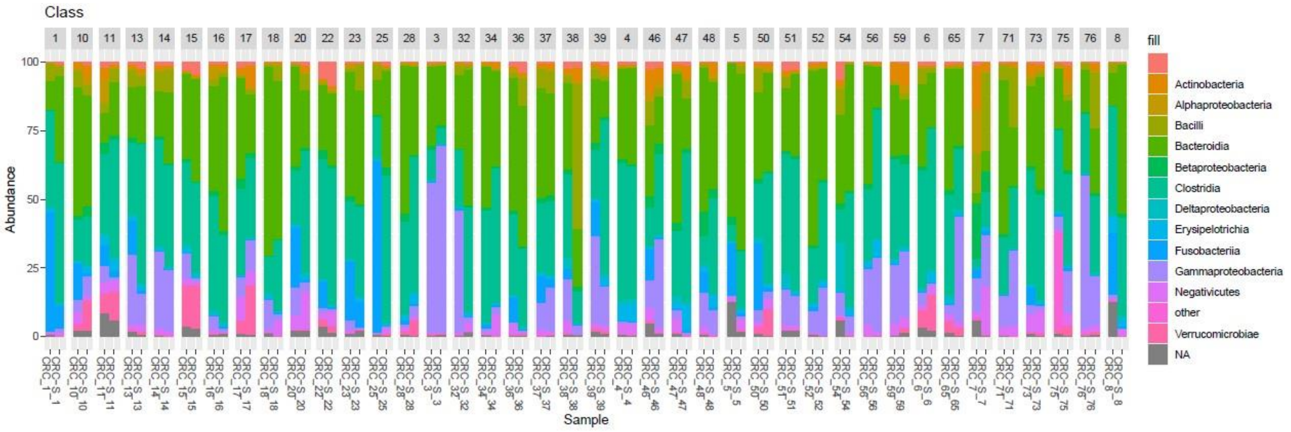

Figure S1. Stacked boxplots of microbial composition at class level of CRC and CRC-S samples.

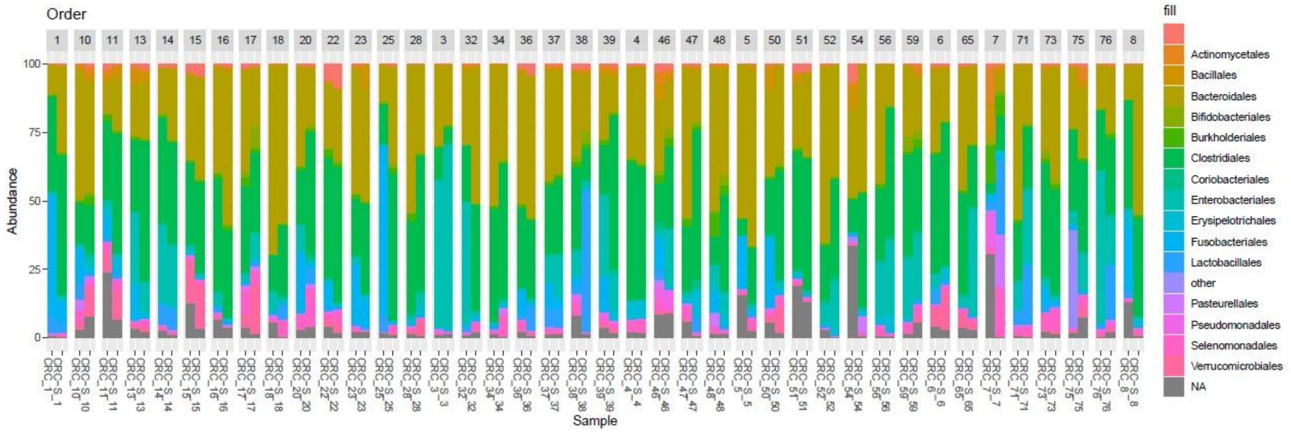

Figure S2. Stacked boxplots of microbial composition at order level of CRC and CRC-S samples.

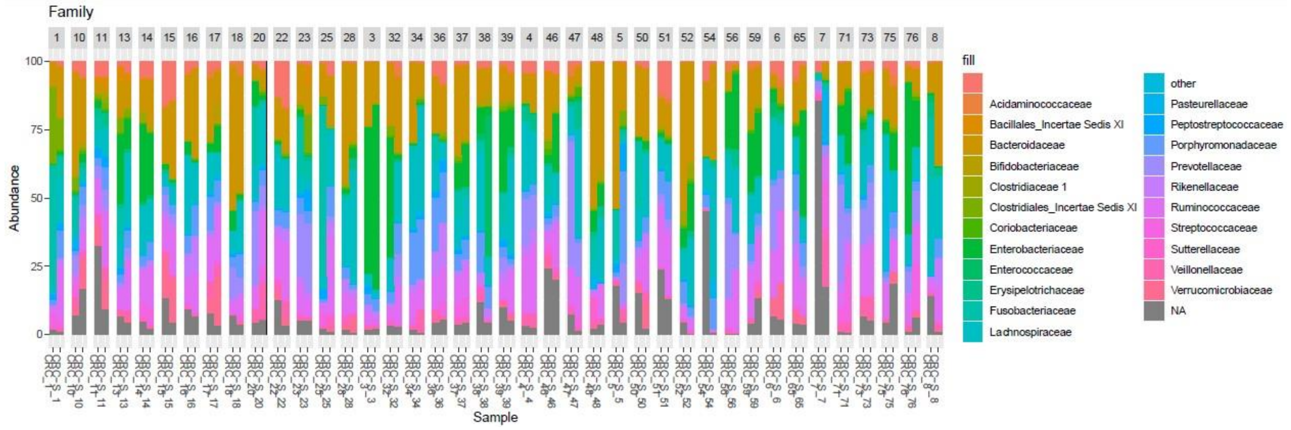

Figure S3. Stacked boxplots of microbial composition at family level of CRC and CRC-S samples.

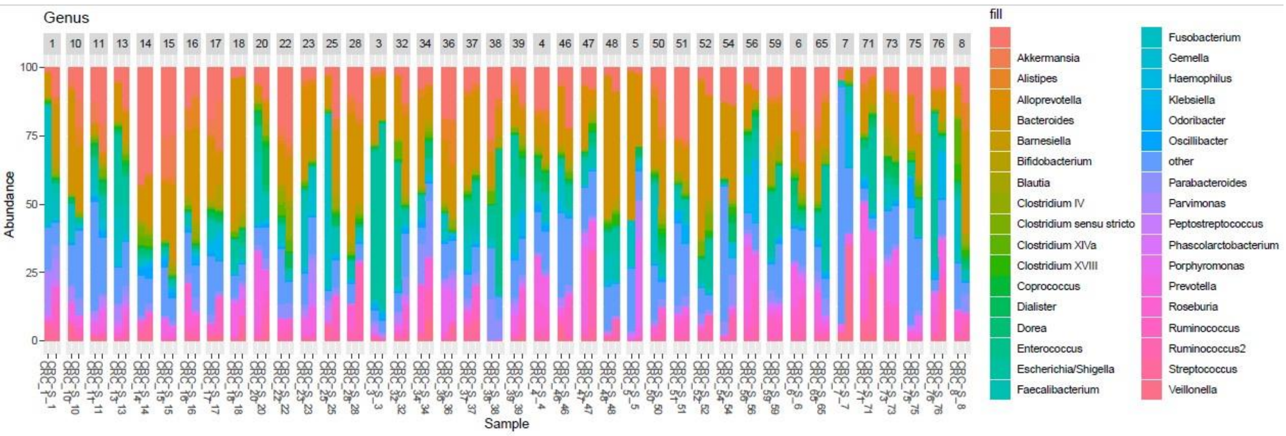

Figure S4. Stacked boxplots of microbial composition at genus level of CRC and CRC-S samples.

DATA AVAILABILITY

The datasets presented in this study can be found in the repository GEO, available online at https://www.ncbi.nlm.nih.gov/geo/query/acc.cgi?acc=GSE163366
